# A Randomised Controlled Trial Comparing Ketamine *versus* Fentanyl for Procedural Sedation in the Emergency Department for Adults with Isolated Extremity Injury

**DOI:** 10.5704/MOJ.2403.015

**Published:** 2024-03

**Authors:** M Srinivasarangan, S Jagadeesh, A Bheemanna, A Sivasankar, A Patil, B Basavaraju, A Sattur

**Affiliations:** 1 Department of Emergency Medicine, JSS Medical College, Mysuru, India; 2 Department of Anesthesiology, Dr Moopen's Medical College, Wayanad, India

**Keywords:** procedural sedation and analgesia, emergency department, ketamine, fentanyl, extremity trauma

## Abstract

**Introduction:**

Alleviating pain and anxiety of patients during procedures is an essential skill for an Emergency Physician (EP). Several sedatives and dissociative agents are used for PSA (Procedural Sedation and Analgesia). In this study, we aimed to compare two drugs that is, ketamine and fentanyl for procedural sedation in adults with isolated limb injuries in the Emergency Department (ED).

**Materials and methods:**

In this prospective, randomised controlled interventional trial, patients aged between 18 to 65 years with isolated extremity injury requiring PSA in the ED were recruited. A total of 200 subjects were included in the study and randomly allocated to either the fentanyl (n=100) or the ketamine (n=100) group. Patients were blinded to the intervention and subsequently premedicated with Midazolam. Following this, they received either ketamine or fentanyl based on the group they were allocated to. Vital signs, including but not limited to the level of sedation, were measured at predetermined time intervals. A Modified Aldrete Score of >8 was used as a criterion for disposition from the ED. Data were collected in a pre-designed proforma. We aimed to compare the effectiveness as well as ascertain the safety profile of the two drugs for PSA in the ED.

**Results:**

There was no significant difference between the two groups when age, gender, mechanism of injury and comorbidities were compared. We found that there was no statistically significant difference between the two groups when blood pressure, respiratory rate and depth of sedation were compared. In both groups, there was a significant decrease in pain on the Numerical Rating Scale (NRS) following drug administration from 8 to 3 (p<0.001). Patients in the fentanyl group had an increased incidence of transient oxygen desaturation (p<0.001). Vomiting was more common in the ketamine group (p<0.001).

**Conclusion:**

PSA is a safe and efficacious procedure for patients undergoing painful procedures in ED. Patients in both the groups maintained hemodynamic stability throughout the procedure. From our study, we were able to conclude that both ketamine and fentanyl are similar in efficacy for PSA in the ED for adults with isolated limb injuries. In addition, no significant cardiovascular adverse events were noted in either group in our study.

## Introduction

Patients with acute pain is a common presenting complaint to the Emergency Department (ED). Across major secondary and tertiary care centres in India, it has been noted that the most common reason for ED visits is trauma and Road Traffic Accidents (RTA). Among the conditions associated with pain, trauma accounts for 24% of the ED visits followed by Abdominal pain (16%) and Chest pain (9%)^1^.

Pain is an unpleasant sensory experience that often has an emotional component. Pain also has a protective component to it as it is an indicator of potential or actual tissue damage. Alleviating pain is a major treatment goal for an Emergency physician (EP). Despite having several methods of pain management, oligoanalgesia is a persisting problem in the ED^2-4^. Effective pain management during the patient’s stay in ED is found to improve patient satisfaction and reduce distress^5,6^.

Not only do patients present to ED with a painful condition, they are also subjected to procedures which can cause both pain and anxiety. Some of the procedures that are performed include closed reduction of fractures, reduction of dislocated joints, placement of urinary catheter, wound wash and suturing etc. It is important to provide analgesia and anxiolysis during these procedures. Some of the methods of pain management performed in ED are intravenous analgesia, local anaesthetic and regional blocks. One of the methods of management of pain during these procedures is by using Procedural Sedation and Analgesia (PSA)^7-9^.

There are five categories of drugs used for PSA which are, sedative-hypnotics, analgesics, dissociative drug, inhalational drug and reversal agents for both opioids and benzodiazepine, that is, naloxone and flumazenil, respectively. Numerous studies have been conducted to compare the efficiency and safety of one drug over the other in the ED for a variety of procedures and patient characteristics. Comparisons between ketamine/midazolam and fentanyl/midazolam made in children undergoing emergency fracture reduction showed that reduction was successful in both groups. However, ketamine/midazolam group was found to be more effective in reducing pain and anxiety in the study population^10,11^. Similar studies done in our part of the world comparing the two drugs could not be found in published literature. Hoping to close this gap in knowledge, we designed the present study in an attempt to compare the effectiveness of ketamine and fentanyl for PSA in adults presenting to the ED of our hospital with isolated limb injuries.

## Materials and Methods

This study is a Randomised Controlled Prospective Interventional trial (RCT) (UTN- U1111-1262-4211) conducted in the Emergency Department (ED) in a tertiary care hospital in India over a period of 18 months (January 2021 to June 2022).

**Fig 1:**
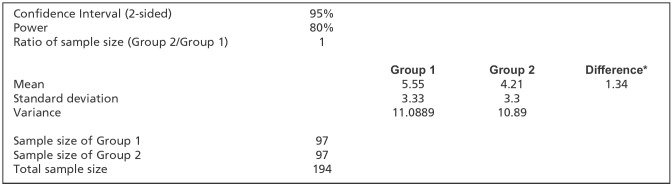
Method used for calculating sample size in our study. Considering the findings from the study done by Robert M Kennedy *et al*^10^, the mean Pain score as per the Facial Affective Scale (FAS) among Fentanyl group and Ketamine group was 5.55+3.33 and 4.21+330, respectively. Based on the data, at 95% Confidence Interval an estimated sample size of 97 in each group was derived based on the above-mentioned formula to adequately power the study to 80%. A total of 100 study samples were included in each group for the purpose of the study.

**Fig 2:**
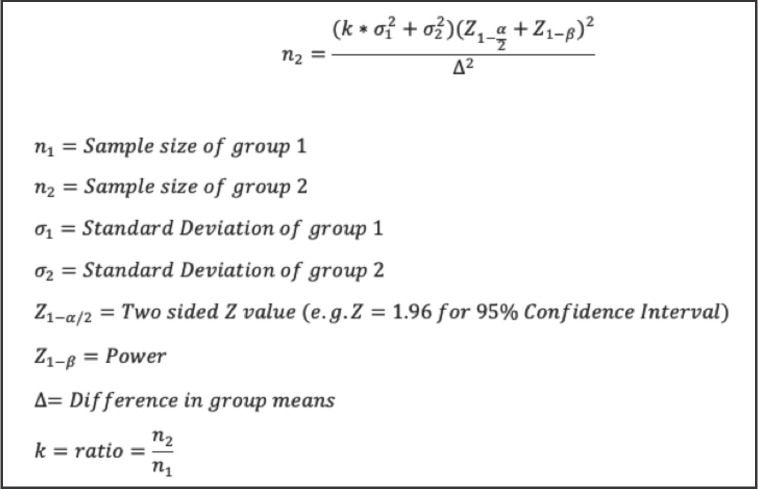
Formula used for calculating the sample size.

In an 1800-bedded tertiary care hospital in India, patients aged between 18 to 65 years with isolated extremity injury requiring procedures such as closed reduction, wound cleaning, wound suturing and immobilisation were included in the study. Institutional Ethics Committee approval was obtained (JSS/MC/PG/5156/2020-21). The study was in accordance with the ethical standards of the institutional ethics committee on human experimentation and with the Helsinki Declaration of 1975, as revised in 1983. Consent was obtained after explaining the risks and benefit of the procedure to the patient in the presence of a witness in the language they understood the best. Sample size was calculated using the method mentioned in the (Fig. 1) and the formula used for the same is depicted below (Fig. 2). Considering the findings from the study done by Robert M Kennedy *et al*^10^, the mean Pain score as per the Facial Affective Scale (FAS) among Fentanyl group and Ketamine group was 5.55+3.33 and 4.21+330, respectively. Based on the above data, at 95% Confidence Interval an estimated sample size of 97 in each group was derived based on the above-mentioned formula to adequately power the study to 80%.

A total of 200 patients fulfilling the inclusion and exclusion criteria were included in the study. Simple random sampling was done and randomisation was done using a paper chit method with arms allocated equally. One hundred patients were allocated to the fentanyl group and 100 patients were allocated to the ketamine group. Data collector and the patient were blinded to the drug being given. Demographic details, medical history and preprocedural vitals were documented along with preprocedural NRS score. The data was collected in a proforma designed for this study.

**Fig 3:**
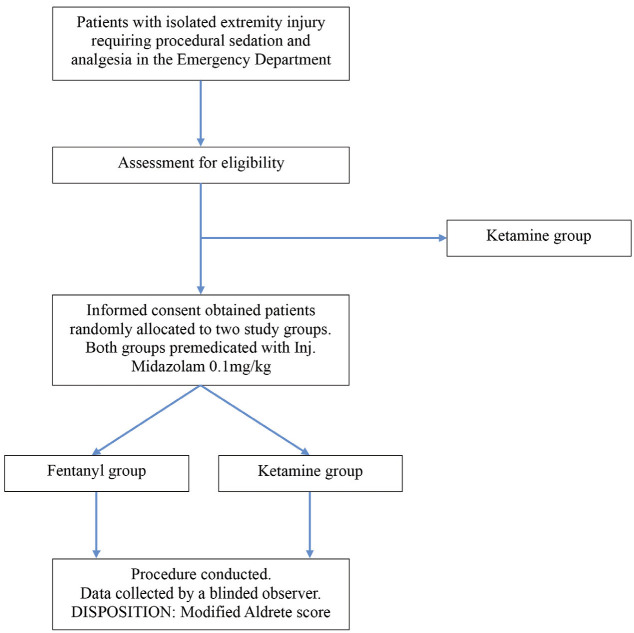
Flow chart depicting the study design.

The procedure was performed in a room fully equipped with monitoring and resuscitation equipment. Patients in both groups were first premedicated with Midazolam </= 0.1mg/kg (Maximum of 2mg/dose) given intravenously over divided doses every 3 minutes till patient became drowsy or was glassy eyed. At least 2 minutes after midazolam administration, the patients in the fentanyl group received </= 0.5mcg/kg (not more than 100mcg/dose) every 3 minutes until a decreased response to verbal or painful stimuli occurred. Maximum dose allowed was 2mcg/kg. Those who were in the ketamine group, </= 0.5mg/kg was given intravenously every 3 minutes until a decreased response to verbal or painful stimuli was observed. Maximum dose was 2mg/kg for ketamine. As the drugs were visibly distinguishable, the clinician performing the PSA was not blinded. During the procedure vitals were monitored every 5 minutes for the first hour; at this time the depth of sedation was assessed using the Ramsay Sedation Scale. After one hour, the wakefulness of the patient was measured using Modified Aldrete Score and vitals were measured every 15 minutes for the next hour. Vitals were then measured every 30 minutes for the 3rd and 4th hour of monitoring. Patients were also monitored for adverse effects of the drugs such as nausea, vomiting, giddiness, hypotension, respiratory depression and emergence delirium. They were treated as per the standard of care for the adverse events if noted. All patients were supplemented with oxygen at 4L/min with a simple face mask. Increased requirement of oxygen, requirement of IV fluids and antiemetics were considered resuscitative measures. During this time, procedures such as fracture reduction, wound wash, wound suturing etc. were done. A Modified Aldrete Score of 8 or more was used as a criterion for disposition from ED. The methodology of the study has been depicted in a flow chart below (Fig. 3). Once baseline sensorium was achieved, patients were requested to answer a questionnaire regarding their satisfaction with the procedural sedation. Similarly, the physicians performing the above-mentioned procedures were given a questionnaire that assessed their level of comfort with the respective drug for PSA.

Inclusion criteria was adults aged between 18 to 65 years with isolated extremity injuries requiring closed reduction, wound cleaning, wound suturing and immobilisation in ED.

Exclusion criteria were (1) haemodynamic instability, (2) allergy to either drug, (3) renal failure, (4) known psychiatric illness on treatment, (5) refused consent to participate in the study, (6) pregnant patients, (positive urine or serum beta human chorionic gonadotropin) (7) patients with an altered perception of pain. Example, history of consumption of alcohol/inebriated at the time of examination.

Data were entered into Microsoft excel data sheet and analysed using SPSS 22 version software [IBM SPSS Statistics, Somers NY, USA]. Categorical data were represented in the form of frequencies and proportions. Chi-square test was used as test of significance for qualitative data. Continuous data were represented as mean and standard deviation. Normality of the continuous data was tested by Kolmogorov–Smirnov test and the Shapiro–Wilk test. Independent t test was used as test of significance to identify the mean difference between two quantitative variables.

Graphical representation of data: MS Excel and MS word were used to obtain various types of graphs such as the Bar diagram, the Pie diagram and the Scatter plots. A p value of <0.05 was considered as statistically significant after assuming all the rules of statistical tests.

## Results

A total of 200 patients were recruited and randomly assigned to the fentanyl and ketamine group (100 each). The patients were analysed for their demographic characteristics. In our study the mean age was 38.26 years (SD-14.04) in the fentanyl group and 35.94 years (SD-14.31) in the ketamine group. Majority of the patients were males in both the groups. There were 83% males in the fentanyl group and 86% males in the ketamine group. Majority of the patients recruited in this study had sustained an injury following RTA (83% and 91% in Fentanyl and Ketamine group, respectively) (Table I).

**Table I:** Table depicting the demographic details, mode of injury and medical history of the patients recruited in the study.

	Fentanyl Group	Ketamine Group
Mean Age (Years)	38.26	35.94
Sex		
Male (%)	83	86
Female (%)	17	14
Mode of injury		
RTA (%)	85	91
Workplace Injury (%)	14	9
Spontaneous Dislocation (%)	1	0
Medical History		
Nil (%)	91	91
Hypertension (%)	3	4
Hypothyroidism (%)	4	2
Diabetes Mellitus	1	2
Diabetes mellitus and Hypertension (%)	0	1
Recurrent Shoulder dislocation (%)	1	0
ECG findings		
Normal (%)	71	74
Sinus tachycardia (%)	29	26

Prior to initiation of procedural sedation, 29% in the fentanyl group and 26% in the ketamine group had sinus tachycardia. An apparent transient increase in the systolic blood pressure in the fentanyl group (139mm Hg) was observed at the 20-minute interval compared to the ketamine group (124mm Hg); however, this was not statistically significant. There was a statistically significant difference between the Systolic Blood Pressure (SBP) measured between the 50th to 75th – minute interval. During this time, the systolic blood pressure was found to be lower in the ketamine group compared to the fentanyl group. The SBP during 50, 55, 60 and 75-minute interval in the fentanyl group is 124, 123, 123, and 123mm Hg, respectively and 120, 118, 118, and 119 in the ketamine group, respectively (p=0.032,0.026,0.010 and 0.043, respectively). There was a transient 3 to 4mm Hg difference between the two groups at the above-mentioned time intervals but this did not require any clinical intervention.

**Fig 4:**
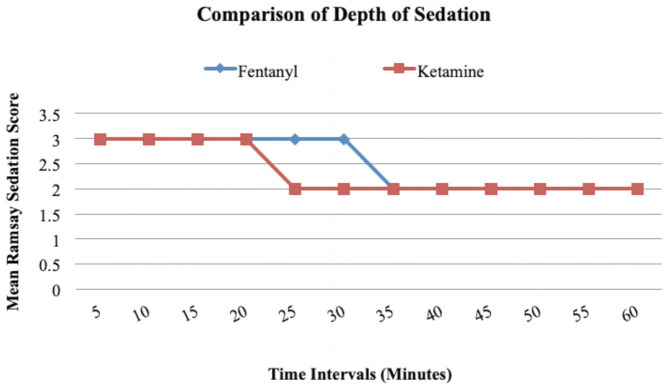
Line diagram for comparison of depth of sedation between two groups at different intervals of time using the Ramsey Sedation Score. Between the 20th to 30th -minute intervals, there was a significant difference between the depth of sedation between the two groups (at 20th minute – p=0.013, at 25th minute- p=0.001 and at 30th minute- p=0.001). Patients in the fentanyl group remained in a higher depth of sedation for 10 minutes longer than those in the ketamine group. At other time intervals, there was no statistically significant difference between the two groups for RSS.

There was no significant difference in respiratory rate, oxygen saturation, heart rate, diastolic blood pressure and mean arterial pressure between the two groups at any interval. Between the 20th to 30th -minute intervals, there was a significant difference between the depth of sedation between the two groups (at 20th minute – p=0.013, at 25th minute- p=0.001 and at 30th minute- p=0.001). Patients in the fentanyl group remained in a higher depth of sedation for 10 minutes longer than those in the ketamine group. At other time intervals, there was no statistically significant difference between the two groups when the Ramsay Sedation Score was compared (Fig. 4).

**Fig 5:**
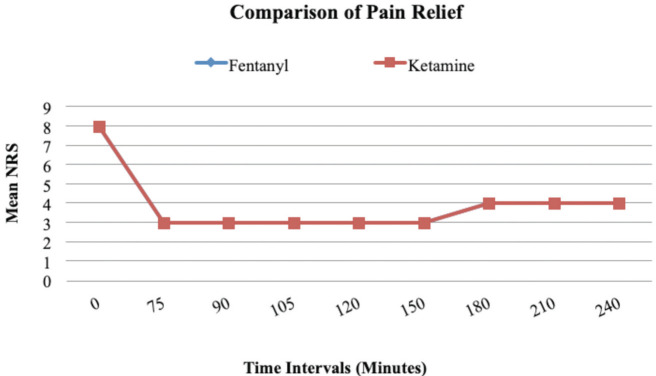
Line diagram comparing pain relief using NRS. There was a significant decrease in the NRS from pre-procedural NRS of 8 to the NRS of 3 at the 75th minute (p = <0.001). However, there was no statistically significant difference of NRS between the two groups at any time interval.

For the NRS (Numerical Rating Scale), there was no statistically significant difference between the two groups at any time interval. The mean NRS had dropped from the mean preprocedural NRS of 8 to a score of 3 at 75th minute since drug administration in both groups. However, when analysed individually, there was a statistically significant fall in NRS in each of the two groups from presentation to 75th minute (p= <0.001) (Fig. 5).

**Fig 6:**
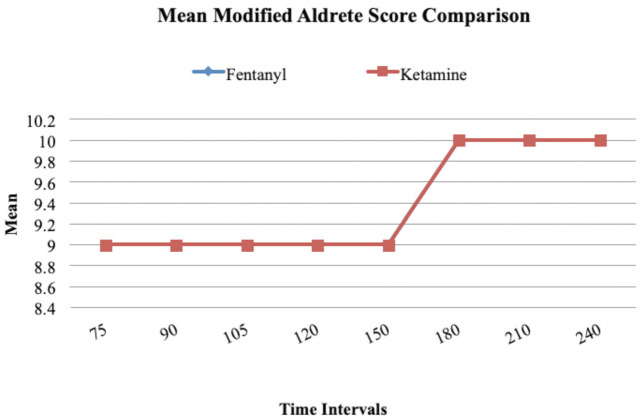
Line diagram comparing Mean Modified Aldrete Score between two groups at different intervals of time. There was no statistically significant difference between the two groups when the Modified Alderete Score was compared.

Modified Aldrete score was used as a criterion for disposition from the ED and it was measured at the 75th minute from drug administration. A score of 8 or more would qualify for disposition. A mean score of 9 was recorded in both fentanyl and ketamine groups at the 75th minute. When the mean modified Aldrete score was compared between the two groups, no statistically significant difference was found (Fig. 6).

The mean time measured between drug administration and starting of procedure was 4 minutes and 4 seconds in the fentanyl group and 4 minutes and 28 seconds in the ketamine group. This difference was not statistically significant (p=0.117). Some patients required repeat dosing of the drug to continue to maintain the desired depth of sedation. Amongst those in the fentanyl and ketamine groups, 12% and 6%, respectively required a repeat dosing of the drug. This difference was not statistically significant (p=0.138). Mean procedure time for the patients in the fentanyl group was 21 minutes and 35 seconds and those in the ketamine group is 24 minutes and 21 seconds. (p=0.117).

The need for resuscitation during PSA for fentanyl and ketamine groups were 13% and 18%, respectively. Resuscitation included the need for antiemetics, IV fluid boluses and need for supplemental oxygen. A statistically significant difference (p=0.046) was found when the two groups were compared for the resuscitation performed with more patients in the fentanyl arm requiring some resuscitation.

## Discussion

PSA (Procedural Sedation and Analgesia) is invaluable in the ED as it provides analgesia and anxiolysis for patients undergoing procedures. The weight-based dosing of sedatives and analgesics allows for better success of procedures, it also reduces the incidence of adverse effects and reduced recovery time. PSA does not have a steep learning curve, procedures can begin within minutes of initiation of PSA and disposition of patients is faster with not all patients requiring hospitalisation.

The study was a randomised controlled interventional trial conducted in an 1800- bedded tertiary care hospital in India. The study recruited patients who presented to ED with isolated limb injuries and required procedures during their stay in the ED, fulfilling the pre-designated inclusion and exclusion criteria.

In our study the median age was 38.26 years (SD-14.04) in the fentanyl group and 35.94 years (SD-14.31) in the ketamine group. Majority of the patients were males in both the groups. There were 83% males in the fentanyl group and 86% males in the ketamine group. This gender distribution was similar to those in the studies done by Kennedy *et al*, Cevik *et al*, Quinn *et al*, Sener *et al*, and Nejati *et al*^10-14^. Majority of the patients recruited in this study had sustained an injury following RTA (Table I). According to WHO, the majority of victims of RTA worldwide were males, approximately 73% and similar findings were confirmed in our study^15^.

There was no significant difference in respiratory rate, oxygen saturation, heart rate, diastolic blood pressure and mean arterial pressure between the two groups at any interval. Studies done by Kennedy *et al*, and Cevik *et al* reported similar findings in their respective studies^10,11^. The study done by Quinn *et al*, which administered ketamine and fentanyl via the intranasal route reported a statistically significant difference in the mean respiratory rates between the two groups with a higher mean respiratory rate observed in the ketamine group (22.2+/- 5.5) as opposed to 18.4 +/1.8 in the fentanyl group, respectively (p=0.05)^12^.

The Ramsay Sedation Score (RSS) was used to quantify the depth of sedation during the first hour of the procedural sedation. RSS was also used in the study done by Nejati *et al* for monitoring depth of sedation^14^. This study achieved an average RSS score of 6 in the fentanyl/midazolam group with a dose of 2mcg/kg. In our study, we targeted a lighter depth of sedation (RSS of 2 – 3). In our study, between the 20th to 30th -minute intervals, there was a significant difference between the depth of sedation between the two groups (at 20th minute – p=0.013, at 25th minute- p=0.001 and at 30th minute- p=0.001). Patients in the fentanyl group remained in a higher depth of sedation for 10 minutes longer than those in the ketamine group (Fig. 4). At other time intervals, there was no statistically significant difference between the two groups for RSS.

The Numerical Rating Scale (NRS) was used to measure pain. The NRS was measured at baseline and then it was serially measured after the first hour. For NRS, there was no statistically significant difference between the two groups at any time interval (Fig. 5). The mean NRS had dropped from the mean preprocedural NRS of 8 to a score of 3, that is, severe pain to mild pain at 75th minute since drug administration in both groups. However, when analysed individually, there was a statistically significant fall in NRS in each of the two groups from presentation to 75th minute (p= <0.001). These findings are similar to the study done by Quinn *et al* where there was no difference of NRS between the two groups after the first hour^12^.

Modified Aldrete score was used as a criterion for discharge from ED. A score of eight or more would qualify for discharge. A score of nine was recorded in both fentanyl and ketamine groups at the 75th minute. When the modified Aldrete score was compared between the two groups, no statistically significant difference was found (Fig. 6). This reemphasised the efficiency of PSA in terms of time utilised for the procedure. With the mean Modified Aldrete Score reaching the target value of 8 or more by the end of the 75th minute, there is a potential for decreasing the time the patient spends in the ED allowing faster disposition.

**Fig 7:**
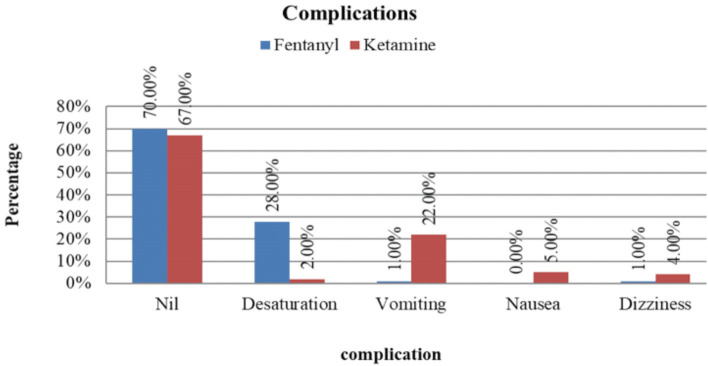
The Bar diagram showing complications that occurred during the conduct of the study. About 70% in the fentanyl group and 67% in the ketamine group had no complications. A total of 5% in the ketamine group reported feeling nauseous. 4% in the ketamine group and 1% in the fentanyl group felt dizziness during the procedure. A total of 28% in the fentanyl group and 2% in the ketamine group had desaturation. When incidence of desaturation was compared between both the groups, there was a statistically significant difference (p=0.0001) between the fentanyl and ketamine groups. The incidence of vomiting was also compared in a similar manner. A total of 22% in the ketamine group had vomiting during the procedure and 1% of those in the fentanyl had vomiting. This difference in incidence of vomiting was statistically significant (p=0.0001).

Desaturation, vomiting, nausea and dizziness were the complications encountered during the study. About 70% in the fentanyl group and 67% in the ketamine group had no complications. A total of 5% in the ketamine group reported feeling nauseous, 4% in the ketamine group and 1% in the fentanyl group felt dizziness during the procedure, 28% in the fentanyl group and 2% in the ketamine group had desaturation. When incidence of desaturation was compared between both the groups, there was a statistically significant difference (p=0.0001) between the fentanyl and ketamine groups. Desaturation following fentanyl administration is a known complication occurring secondary to dose-dependent respiratory depression. The incidence of vomiting was also compared in a similar manner. A total of 22% in the ketamine group had vomiting during the procedure and 1% of those in the fentanyl had vomiting. This difference in incidence of vomiting was statistically significant (p=0.0001) (Fig. 7). Ketamine associated vomiting is a known non-dose dependent complication^16^.

Patients undergoing procedural sedation and analgesia as part of this study were given a questionnaire prior to discharge which included various questions about their experience during the process. When asked about their willingness for the same type of drug in the future, 80% in the fentanyl group and 82% in the ketamine group agreed with the statement. A total of 1% in the fentanyl group were not willing to accept the same drug in the future, 1% of the patients in the fentanyl group reported feeling pain during the procedure as opposed to none in the ketamine group. When asked if they felt satisfied with the care given, 79% in the fentanyl group and 75% in the ketamine group said they agreed with the statement.

Physicians and surgeons performing procedures such as wound wash, wound suturing and closed reduction of fractures were asked about their experience with the procedural sedation. When asked about their satisfaction of patients’ co-operation, 97% in the ketamine group and 90% in the fentanyl group were completely satisfied. A total of 79% in the fentanyl group and 77% in the ketamine group were completely satisfied with the hemodynamic stability maintained after the administration of the drug. Regarding the desired muscle relaxation, 77% in the fentanyl group and 81% in the ketamine group were completely satisfied. They were asked about acceptance for similar procedural sedation in the future. A total of 92% in the fentanyl group and 100% in the ketamine group had acceptance for similar drug for their subsequent procedural sedation. There was a significant difference between the two groups (p=0.011) with better acceptance trends for ketamine. The reason for better acceptance in the ketamine group is hypothesised to be better hemodynamic stability, lesser incidence of hypoxia and preservation of reflexes which allows early discharge. However, our study did not prove superiority of one drug over the other for most of these reasons other than hypoxia which was less in the ketamine group compared to the fentanyl group.

There was an increased incidence of vomiting and nausea with ketamine and transient oxygen desaturation with fentanyl. Serious complications did not occur during the procedure. There was also a significant decrease in the NRS after drug administration. Both drugs had similar analgesic effects. From our study we were able to infer that both ketamine and fentanyl were similar in terms of efficacy for PSA for isolated limb injuries in the ED.

## Conclusion

Alleviating pain and anxiety in patients during procedures is an essential skill for an EP. PSA is one such method that provides analgesia and anxiolysis during procedures that are performed in the ED. The mean time between drug administration and beginning of procedure was less than seven minutes in both groups. This makes PSA using both ketamine and fentanyl a quick and effective option to perform procedures in ED. There was a significant decrease in the NRS following drug administration in both groups from a score of 8 to 3 in both the groups (p=<0.01). This demonstrates that both drugs were similarly effective in providing analgesia. Patients in both groups maintained hemodynamic stability during the procedure. There were increased incidences of vomiting and nausea in the ketamine group and a few patients in the fentanyl group had desaturation. However, none of the patients suffered serious complications during the course of the study. Therefore, we were able to demonstrate the safety of the two drugs for PSA in the ED. From this study we were able to conclude that both ketamine and fentanyl had similar efficacy for PSA in the ED for adults with isolated limb injuries. Superiority of one drug over the other could not be demonstrated in our study.
